# Enhancing Sustainability: Jute Fiber-Reinforced Bio-Based Sandwich Composites for Use in Battery Boxes

**DOI:** 10.3390/polym15183842

**Published:** 2023-09-21

**Authors:** Mina Arya, Else-Marie Malmek, Thomas Koch Ecoist, Jocke Pettersson, Mikael Skrifvars, Pooria Khalili

**Affiliations:** 1 Faculty of Textiles, Engineering and Business (Swedish Centre for Resource Recovery), University of Borås, 510 90 Borås, Sweden; mina.arya@hb.se (M.A.); mikael.skrifvars@hb.se (M.S.); 2 Juteborg AB, 426 79 Västra Frölunda, Sweden; else-marie.malmek@malmeken.se; 3 Ecoist AB, 262 72 Ängelholm, Sweden; 4 RISE Research Institutes of Sweden, 431 53 Mölndal, Sweden; jocke.pettersson@ri.se

**Keywords:** bio-based sandwich composites, composite laminate, jute fiber, plasma treatment, mechanical behavior

## Abstract

The rising industrial demand for environmentally friendly and sustainable materials has shifted the attention from synthetic to natural fibers. Natural fibers provide advantages like affordability, lightweight nature, and renewability. Jute fibers’ substantial production potential and cost-efficiency have propelled current research in this field. In this study, the mechanical behavior (tensile, flexural, and interlaminar shear properties) of plasma-treated jute composite laminates and the flexural behavior of jute fabric-reinforced sandwich composites were investigated. Non-woven mat fiber (MFC), jute fiber (JFC), dried jute fiber (DJFC), and plasma-treated jute fiber (TJFC) composite laminates, as well as sandwich composites consisting of jute fabric bio-based unsaturated polyester (UPE) composite as facing material and polyethylene terephthalate (PET70 and PET100) and polyvinyl chloride (PVC) as core materials were fabricated to compare their functional properties. Plasma treatment of jute composite laminate had a positive effect on some of the mechanical properties, which led to an improvement in Young’s modulus (7.17 GPa) and tensile strength (53.61 MPa) of 14% and 8.5%, respectively, as well as, in flexural strength (93.71 MPa) and flexural modulus (5.20 GPa) of 24% and 35%, respectively, compared to those of JFC. In addition, the results demonstrated that the flexural properties of jute sandwich composites can be significantly enhanced by incorporating PET100 foams as core materials.

## 1. Introduction

The use of natural fiber-reinforced composites is rapidly gaining popularity as a promising and environmentally friendly alternative to traditional synthetic fiber composites. Natural fibers offer numerous advantages, including their lightweight nature and low density (e.g., natural fibers have a density of 1.3 g/cm^3^ compared to 2.5 g/cm^3^ for glass fiber), resulting in improved stiffness-to-weight ratios for composite materials [1,2,3,4,5]. Jute fiber stands as one of the most crucial agrofibers in the industry. Its affordability and good availability make it a favorable choice compared to other natural fibers. The economical and nonabrasive characteristics of jute fiber enable the incorporation of high filling levels, leading to significant cost savings in composite manufacturing [6]. Traditionally, jute fibers were primarily used for manufacturing jute ropes, twines, sacks, and hessian cloths. However, in recent years, the applications of jute fibers have expanded beyond these traditional uses. The automotive sector, in particular, has started to embrace the benefits of jute fibers for various components and parts such as interior door panels, seatbacks, and trunk liners [7,8]. However, natural fibers possess a significant drawback due to their highly hydrophilic nature. This hydrophilicity hinders their application as reinforcements in composites, as the poor resistance of natural fibers to moisture results in incompatibility and inadequate wettability with hydrophobic polymer matrices [9]. To overcome this limitation, researchers have extensively investigated various chemical and physical modification methods [10,11] for natural fibers to enhance their compatibility with hydrophobic polymer matrices. Shcherbakov et al. [12] investigated the impact of microwave modification on both the structure and imperfection degree of multi-walled carbon nanotubes (MWCNTs). In another study, the effect of the low-temperature argon plasma treatment of fabrics based on polyester fibers, in order to subsequently control polycondensation synthesis of anion-exchange composite heterogeneous membranes, has been presented by Terin et al. [13]. Cyras et al. [14] studied the effect of chemical treatment on the mechanical properties of starch-based blends reinforced with sisal fiber. A systematic investigation by Bledzki et al. [15] was conducted to examine the impact of mercerization treatment on the structure and properties of bast fibers, specifically hemp yarn. The study also explored the effects of fiber pretreatments, including mercerization and the application of MAH-PP coupling agents, on the properties of unidirectional EP (epoxy) and PP (polypropylene) model composites using flax and hemp fibers. In another attempt, PLA/fiber composites were prepared by Spiridon et al. [16] using cellulose fibers obtained through the organosolv process. To enhance the adhesion between the fibers and the polymer matrix, the cellulose fibers were chemically treated using stearoyl chloride and enzymatically treated using laccase. Non-chemical modifications on the fiber surface, were applied to enhance mechanical performance of the composite laminates without causing adverse environmental impact. Ivanovska et al.’s [17] investigation focused on analyzing the impact of air plasma treatment (at 150 or 300 Hz) on the wettability of raw jute fabric. This assessment was carried out by measuring wetting time and capillary rise height. Their findings suggested that both plasma treatments led to enhanced wettability of the jute fabrics. Deus et al. [18] reported that drying the fibers to reduce absorbed water prior to their incorporation into the polyester matrix has a significant impact on the wetting process between the fiber and the polymeric matrix. This treatment improves the interaction between the fiber and the matrix, leading to enhanced fiber-to-matrix adhesion. Plasma treatment, known as a dry technique with low environmental impact, can be also employed to create hydrophobic surfaces. Ricciardi et al. [19] investigated the impact of a one-step sulfur hexafluoride (SF6) plasma treatment on the low-velocity impact behavior of basalt/epoxy composites. They concluded that the treated samples exhibited confined damage, as well as lower absorbed energy and indentation depth, compared to the unmodified composites. This confirms that the plasma treatment effectively modifies the surface properties, resulting in reduced visible impact damage to the composites.

Limited research has been conducted on the utilization of jute fabrics as both facing material used in panels and sandwich composites. Additionally, to the best of the authors’ knowledge, the use of bio-based UPE (unsaturated polyester) in sustainable sandwich bio-composites with PVC or PET as core material, has not been previously investigated. This study involved the manufacturing of composite laminates and bio-based sandwich composites using jute fabrics, with bio-based UPE as well as PVC and PET as core materials. The bio-based UPE used in the manufactured composites, contained approximately 30% biological content and was designated as recyclable, similar to other synthetic PEs. This UPE grade was chosen for its high impact resistance, good processability, and medium stiffness, making it suitable for part production. The investigation focused on studying the effects of treated jute fabrics into the bio-based UPE in terms of tensile, flexural, and interlaminar shear tests properties of the composite laminates. Furthermore, the bending behavior of the different sandwich bio-based composites was also examined. The purpose of this project is to demonstrate the feasibility of substituting conventional reinforcement materials like glass and carbon fiber with a more sustainable alternative, jute fiber, while maintaining or even improving the functional properties of the composite materials. The aim is to highlight the potential of jute fiber as an eco-friendly and viable option for reinforcement, promoting sustainable practices in the composite industry.

## 2. Materials and Methods

### 2.1. Materials

Juteborg Sweden AB supplied 0/90 plain woven jute fabrics. The jute fabrics had a surface weight of 500 g/m^2^ and 260 g/m^2^ and a thickness of 2.2 mm and 0.8 mm, respectively. The bio-based unsaturated polyester, which contained up to 30% bio-based content, was purchased from Scott Bader Company, Wollaston, UK. The Crystic^®^ resin 807PA had a viscosity of 1.6 Pa·s at 20 °C and specific gravity of 1.1 g/mL at 25 °C. The catalyst employed was MEKP 50% with a moderate reactivity feature. It was recommended to incorporate this catalyst into the resin at a weight ratio of 2% and utilize a mechanical stirrer with low shear. PVC and PET (two kind of PET were applied, PET70 and PET100) were used as core material for the sandwich composite, and PVC and PET100 were supplied by DIAB AB, Helsingborg, Sweden, and PET70 was supplied by Armacell,, Liège, Belgium. The PVC, PET70, and PET100 possessed a density of 48 kg/m^3^, 78 kg/m^3^, and 105 kg/m^3^, respectively, and a thickness of 10 mm for all core materials. To prevent moisture absorption during the composite processing, the jute fabrics were subjected to drying in a convection oven for 16 h at a temperature of 60 °C.

### 2.2. Low-Pressure Plasma Treatment

In this study, a LP (low pressure) plasma system was utilized for surface treatment. Oxygen plasma was employed to treat the jute fabrics at plasma powers of 320 W for a duration of 90 s. The LP plasma equipment used in this work was the PICO type LF plasma equipment provided by Diener Electronics GmbH + Co., Ltd., Ebhausen, Germany. The plasma generation process began by creating a low pressure within the chamber using a vacuum pump (Trivac, Dresden, Germany). Once the pressure reached approximately 0.23 mbar, oxygen was introduced into the chamber up to approximately 0.70 mBar. The plasma was then switched on at 320 W and held for 90 s. After the treatment, the fibers were kept in ambient atmosphere for about 1 h until they were used in the process for making laminates. Weight loss of the fibers during plasma treatment was in the range of 2–3% and can be explained by the possibility that drying of the fibers may have occurred during the treatment.

### 2.3. Preparation of Laminate and Sandwich Composites Using Vacuum Resin Infusion

The fabrication of composite laminates was carried out using the vacuum-assisted resin infusion process. The process involved stacking of different layers of jute fabrics (which depends on the type of the laminates, according to Table 1) on a glass mold, forming a plate with a desired thickness for each laminate (as shown in Table 1). The fiber mass content in the composites was shown in Table 1. To facilitate easy removal of the composite plate after curing, a mold-releasing sheet was placed under the fiber layers. A highly permeable medium was sequentially placed over the fiber surface, and the entire setup was enclosed in a vacuum bag, which was tightly sealed onto the mold. Under vacuum pressure, a polyester resin mixed with the curing agent was injected into the fiber layers. Vacuum infusion started at −1 bar and increased to −0.8 bar prior to complete filling of mold to reduce out-gassing. No degassing of resin prior to infusion was needed. The composite was allowed to cure overnight at 60 °C. After the initial curing period, the composite plates were carefully removed from the glass mold. To complete the curing process, they were subjected to post-curing in a hot air oven at 60 °C for 24 h [20]. This step ensured further strengthening and stabilization of the composite structure. Additionally, composite laminates incorporating dried and plasma-treated fibers (DJFC and TJFC), were prepared using a similar fabrication method. Dimensions of the fabric sheet were 600 mm × 100 mm. Computational numerical control machine (CNC) was utilized for precise cutting of the mechanical test samples.

The jute bio-based unsaturated polyester sandwich composites with the PET (PET70 and PET100) and PVC cores were fabricated by placing a layer of jute fabric on both the top and bottom of the core material, and then, the mentioned steps for the fabrication of the composite laminate were repeated.

### 2.4. Characterization

#### 2.4.1. Tensile Test

Tensile behavior of the dog-bone jute fiber bio-based composite laminates (MFC, JFC, DJFC, and TJFC) were investigated in accordance with the EN ISO 527-4 (type 1B specimen) [21] standard using a Tinius Olsen H10KT (Horsham, PE, USA) testing machine. To measure strain, a 100R mechanical extensometer was attached to the specimens. The rate of loading during the tests was set to 2 mm/min, and the load cell applied was 5 kN. The gauge length used for tests was 50 mm, while the initial distance between the grips was 115 mm. A CNC-milling machine (CNC-STEP GmbH & Co. KG, Geldern, Germany) was utilized for all the composite specimens’ cutting. To ensure reliability and obtain an average result, a total of eight identical specimens were tested. Before conducting any testing, the samples were subjected to a conditioning period of 24 h in a humidity chamber. During this time, the samples were kept at a temperature of 23 °C and a humidity level of 50%. Conditioning the samples under standardized environmental conditions ensures consistency and allows for accurate and reliable test results.

#### 2.4.2. Flexural Test

The bio-based composite laminates (MFC, JFC, DJFC, and TJFC) were evaluated under three-point bending tests according to the BS EN ISO 14125 standard [22]. The tests were conducted using a Tinius Olsen H10KT universal testing instrument. The purpose was to compare the flexural properties of the samples. During the tests, a crosshead speed of 5 mm/min was maintained, and the span length between the supports was set at 64 mm. The samples had a length of 80 mm and a width of 20 mm. A load cell with a capacity of 250 N was connected to the testing equipment. Prior to the testing, all specimens were conditioned for 24 h at a humidity level of 50% and a temperature of 23 °C. To ensure reliability, a total of seven identical specimens were tested. The obtained test results were analyzed, and the average and standard deviation values were reported for each sample type. This comparison allowed for an assessment of the flexural properties of the different bio-based composite laminates and sandwich composite samples.

Sandwich flatwise three-point flexural tests were performed using a Tinius Olsen H10KT (Horsham, PE, USA) universal testing machine equipped with a 5 kN load cell, following the ASTM C 393 [23] standard. Rectangular specimens were utilized for the tests, the specimen dimensions were 120 mm (length) × 30 mm (width) × 10 mm (thickness), with a span length of 70 mm. The test speed was set at 5 mm/min. Prior to testing, the specimens were conditioned at a temperature of 23 °C and a relative humidity of 50% for 24 h. At least three specimens were tested for each group of specimens to ensure statistical significance and reliable results.

#### 2.4.3. Interlaminate Shear Test

In addition to tensile and flexural loads, shear loads also play a significant role in the behavior of laminate components during their practical usage. Interlaminar shear strength (ILSS) testing was conducted using the ILSS test fixture in accordance with ASTM D 2344. The testing machine mentioned earlier was used for this purpose. For the ILSS test, a small beam with a length of 20 mm and a rectangular cross-section (width is twice the thickness) was utilized, and the span length between the supports was set at 16 mm. The beam was subjected to three-point bending. The loading was applied at a rate of 2 mm/min. During the test, the specimen experienced both normal (bending) and transverse shear stresses due to the downward force exerted by the loading cylinder. By employing a short-beam configuration, it was assumed that the beam’s length was short enough to minimize bending stresses [24]. This aimed to induce interlaminar shear failure, characterized by cracking along a horizontal plane between the laminae. The force applied at the point of failure was recorded, and the stresses were calculated using the equation Fabs = 0.75 × P_m/(b × h). Here, Fabs represents the interlaminar shear strength in N/mm², Pm denotes the breaking load in N, and b and h represent the width and depth of the specimen in mm, respectively. A span-to-depth ratio of 4:1 was chosen for the test setup. To ensure reliability and obtain an average result, eight identical specimens were tested. The ILSS values from these tests were averaged to determine the interlaminar shear strength for each stacking sequence.

#### 2.4.4. Digital Imaging Microscope Analysis

To assess the interfacial adhesion between the core and the composite skin in the sandwich panel, as well as between the fabric layers and the bio-based UPE in the jute composite laminates, digital imaging microscope tests were conducted. The samples were examined using a Nikon eclipse LV150 N microscope equipped with an advanced optical system and digital imaging capabilities. Cross-sectional images of the samples were captured without any surface coating, allowing for detailed analysis of the interfacial adhesion at a microscopic level.

## 3. Results and Discussion

### 3.1. Tensile Properties of Composite Facings

**Figure 1:**
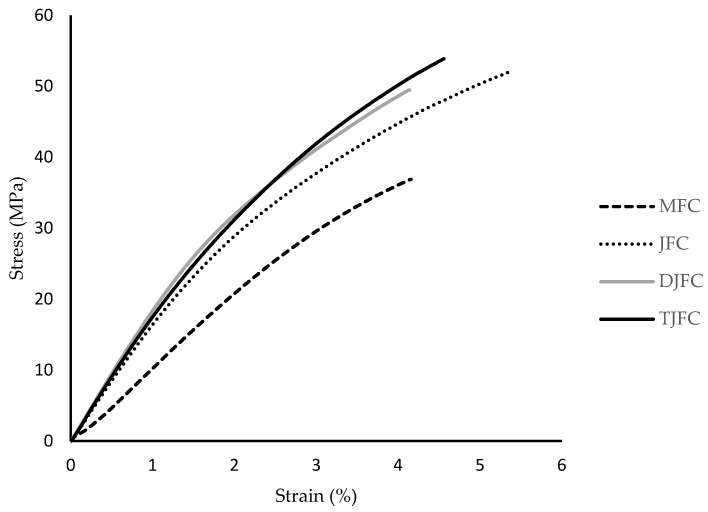
Tensile stress-strain diagram of the composite laminates.

**Figure 2:**
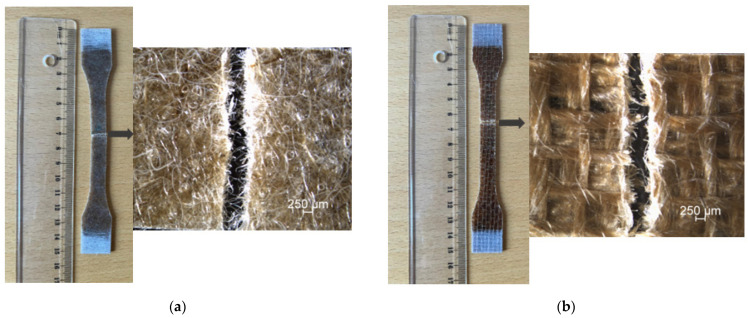

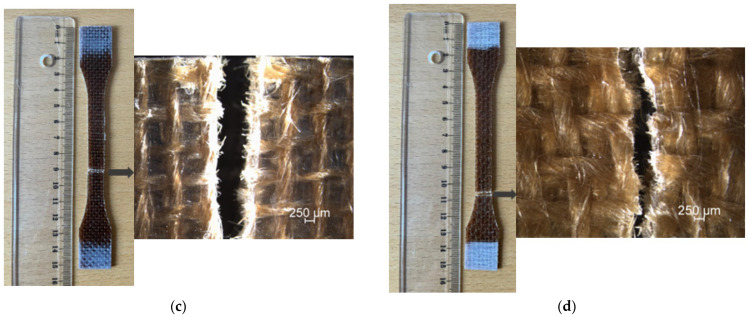
Microscopic and macroscopic images of the broken dog-bone tensile test samples for (**a**) MFC, (**b**) JFC, (**c**) DJFC, and (**d**) TJFC.

The tensile strength, breaking elongation, and modulus values of mat fiber composite (MFC), jute fiber composite (JFC), dried jute fiber composite (DJFC), and plasma-treated jute fiber composite (TJFC) are given in Table 2. According to the results in Table 2, the tensile strength and modulus values of unwoven mat jute fiber composite 18 wt.% (MFC) is found to be 35.66 MPa and 3.63 GPa, respectively. An increase in the tensile strength (49.07 MPa) and modulus (6.12 GPa) of 27% and 40% was found for the 0/90 plain woven fiber composite 32 wt. % (JFC). This illustrates the reinforcement effect of woven fabrics in the composite as the jute fibers’ content increased. Drying of the composite was seen to improve the mechanical properties of the bio-based composite (DJFC) in comparison with those of jute fiber composite (JFC) laminate. Plasma treatment, another non-chemical modification on the fiber surface, was applied to enhance mechanical performance of the composite laminates without causing adverse environmental impact. The increase in the tensile strength and modulus values for plasma-treated jute fiber composite (TJFC) is a proof of the positive effects of plasma treatment on mechanical performance of resulting composites that led to an improvement in modulus (7.17 GPa) by 14% as compared to that of JFC. Breaking elongation of the specimens (considering standard deviation) decreased in the order MFC < JFC ≈ TJFC < DJFC. This trend can be attributed to the formation of a weak bonding between the bio-based thermoset resin and the weak spots of the untreated (natural) jute fibers as well as superior interfacial adhesion of plasma-treated jute fiber composite to the hydrophobic polymer matrix (polyester resin). The reason could be that the tensile behavior of composite fibers is influenced by several factors, including the quality of fiber–matrix coupling, matrix toughness, and the presence of local stress concentration caused by the interaction between fibers and matrix heterogeneities [25]. Sinha et al. [26] investigated the effects of physical treatment on the morphology, wettability, and fine structure of fibers, and its influence on the interfacial adhesion of thermoset composites reinforced with jute fibers, and they found that the plasma treatment led to the development of hydrophobicity in the fibers, as evidenced by increased contact angles with water. This hydrophobicity may be attributed to a decrease in phenolic and secondary alcoholic groups, as well as the oxidation of key structural components such as lignin and hemicelluloses, as observed with FT-IR analysis. Gibeop et al. [27] examined the impact of plasma treatment on the mechanical, morphological, and interlaminar shear strength (IFSS) of jute/PLA composites. Furthermore, the IFSS and hardness of the composites increased with plasma treatment. Based on the findings of this study, the authors concluded that plasma treatment is an effective and environmentally friendly alternative to chemical methods for improving the properties of jute/PLA composites. Plasma treatment was found to induce changes in the morphology of the jute fiber and the fracture surface of the composites with PLA. The tensile strength, modulus, and flexural strength of the plasma-treated jute fiber composites showed slight improvements compared to untreated (UT) and alkali-treated (AT) composites. This enhancement in mechanical properties can be attributed to the heat and etching effects of the plasma treatment, resulting in a rougher fiber surface that promotes better interlocking between the fiber and matrix. Wang et al. [28] investigated the impact of various treatment including acid pretreatment, alkali pretreatment, and scouring of jute fiber on mechanical and physical properties of jute fiber composites. They found that treated jute fiber composites demonstrate better performance in terms of tensile strength, void fraction, elongation at break, and interfacial matrix–fiber bonding. Figure 1 shows the tensile stress–strain diagrams of the different composite laminates. A linear behavior at low level of loading before reaching the plastic zone can be seen for all the composite laminates, followed by a steadier increase in load, and consequently, the final failure of composite samples at low strain values. The trend implies that the specimens indicate a failure behavior of brittle type with smooth fracture surface structure, as the macroscopic and microscopic appearances of the broken tensile test specimens are a proof of this pattern (Figure 2).

### 3.2. Flexural Properties of Composite Facings

**Figure 3:**
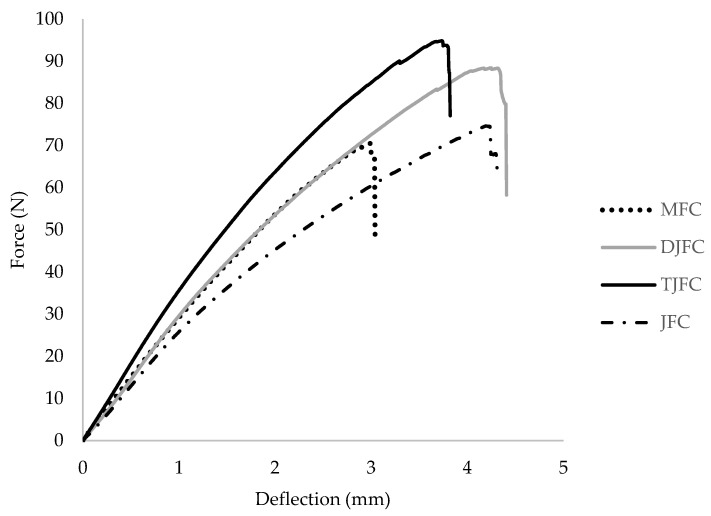
Comparison of force–deflection responses of MFC, JFC, DJFC, and TJFC.

Flexural strength and modulus values of MFC, JFC, DJFC, and TJFC laminates are given in Table 3. Figure 3 displays the force–deflection diagrams of various composite laminates. The flexural strength of MFC and JFC specimens are similar (71.26 MPa for MFC and 70.78 MPa for JFC), and this similarity indicates comparable strength and deformation characteristics of the fiber composites when subjected to bending loads. The flexural strength of DJFC (87.08 MPa) and TJFC (93.71 MPa) were improved by 19% and 24% as compared to that of JFC, due to positive impact of jute fiber treatment. It is clear from Table 3 that this improvement is more significant in TJFC specimen. Considering the modulus values, it was shown that flexural modulus followed the same trend as Young’s modulus (TGFC > DJFC > MFC > JFC) except for MFC and JFC, which are similar (4.12 GPa for MFC and 3.38 GPa for JFC). The flexural force–deflection curves exhibited an initial linear region followed by a nonlinear behavior leading up to fracture for all specimens.

### 3.3. Interlaminar Shear Test Properties of Composite Facings

**Figure 4:**
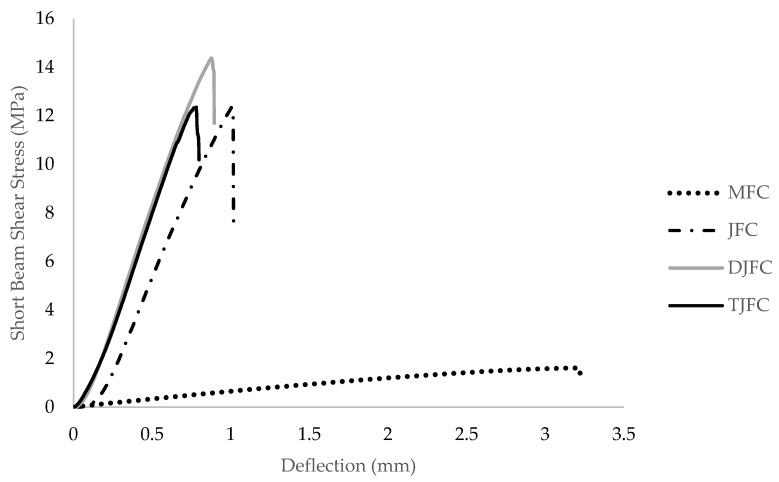
Short-beam shear stress–deflection diagram of the composite laminates.

The short-beam shear stress–deflection diagrams of interlaminar shear testing for various composite laminates are presented in Figure 4. As observed from the diagrams, trends are similar for tensile tests. In short-beam shear tests, the occurrence of maximum shear stress differs from what is predicted by homogeneous beam theory. While homogeneous beam theory states that the maximum shear stress appears at the neutral plane where normal stresses are zero, in reality, the maximum shear stress is found in an area where other stresses may exist. This leads to a combination of failure modes, including fiber rupture, micro buckling, and interlaminar shear cracking, as described by Mallick [29]. Interlaminar shear failure may not necessarily occur at the laminate midplane, making it challenging to achieve pure shear failure along the interface. Consequently, interpreting data from short-beam tests becomes difficult. Interlaminar shear test properties of the jute fiber composites are given in Table 4. DJFC laminates demonstrated an average interlaminar shear stress (ILSS) value of 14.11 MPa (shown in Table 4), which is in agreement with the results obtained previously. For example, Sabeel Ahmed et al. [20] reported an average interlaminar shear stress value of 13.9 MPa for jute laminates. The MFC is found to have lowest interlaminar shear strength of 7.57 MPa among all the laminates. The interlaminar shear strength values are similar for JFC (12.51 MPa) and TJFC (12.63 MPa). It is important to note that the interlaminar shear strength primarily depends on the properties of the matrix and the interfacial strength between the fibers and the matrix, rather than the properties of the fibers alone. Improving ILSS can be achieved by increasing the matrix’s tensile strength and the volume fraction of the matrix [20].

### 3.4. Flexural Properties of Sandwich Panels

**Figure 5:**
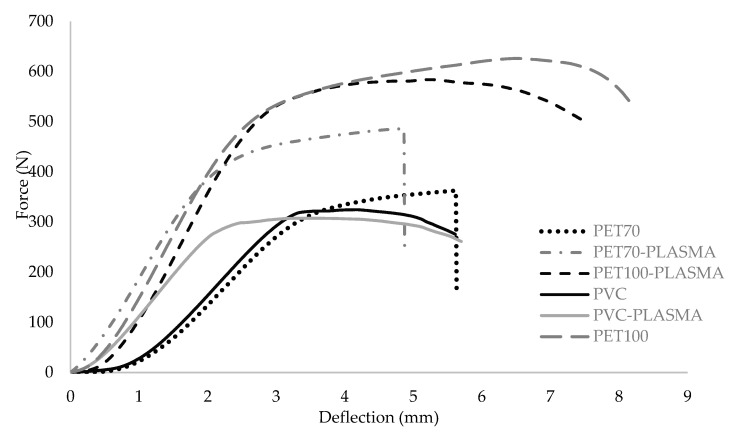
Force–deflection behavior of jute fiber sandwich panels.

**Figure 6:**
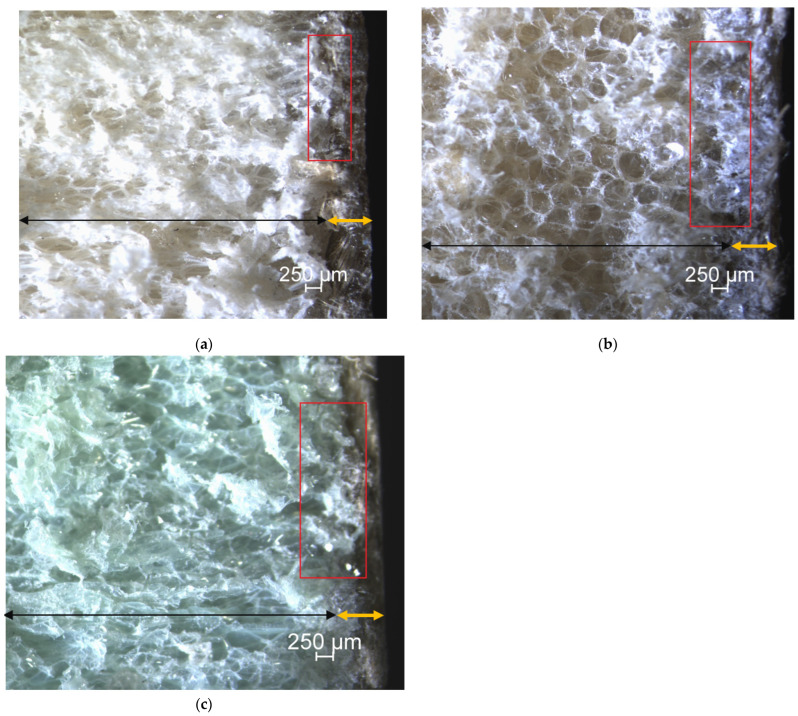
Microscopic images of the interface: (**a**) between the composite skin and PET100 as the core, (**b**) between the composite skin and PVC as the core, and (**c**) between the composite skin and PET70 as the core.

Table 5 presents the flexural properties of PET70. PET70-PLASMA, PET100, PET100-PLASMA, PVC, and PVC-PLASMA jute fiber-reinforced sandwich composites. Additionally, Figure 5 illustrates the force–deflection diagrams of various sandwich composites. The results in Table 4 show that the maximum force at failure (max force) decreases in an order of PET100 > PET100-PLASMA > PET70-PLASMA > PET70 > PVC > PVC-PLASMA. The smallest “max force” is 308.83 N for PET100 compared to 607.78 N for PVC-PLASMA, which demonstrates the highest “max force”. Facing ultimate stress (FUS) follows the same order as max force. The highest FUS of 32.23 MPa was obtained for PET100 compared to 18.02 for PVC-PLASMA. The core shear ultimate stress (CSUS) ranges from 0.92 MPa for PET100 to 0.51 MPa for PVC-PLASMA and decreases in the same order (PET100 > PET100-PLASMA > PET70-PLASMA > PET70 > PVC > PVC-PLASMA) as max force and FUS. For the maximum deflection, the highest amount was 7.69 mm for PET100 compared to 5.86 mm for PVC. This is likely attributed to the superior adhesion between natural jute fibers and bio-based PET100 (the yellow and black arrows indicate the direction and composition of the facing and core within the cross-section of the panels, respectively, depicted in Figure 6) as compared to other core materials. The results in Table 5 show that the highest flexural rigidity/width of 433,785 N mm obtained for PET70-PLASMA and PET100-PLASMA compared to 331,000 N.mm for PVC. After conducting the tests, the samples were analyzed, revealing that all the sandwich composite samples remained intact without any breakage. The identified fracture mode was represented by the presence of micro-cracks on the compression (top) sides of the samples. Figure 6 depicts the microstructure of the sandwich composites. The images show favorable interfacial adhesion between the PET100 (core) and the facing (a) compared to PVC (b) and PET70 (c) counterparts as indicated by the red rectangular. As shown in Figure 6a, a cohesive interaction between the fibers and the matrix, along with a minor infiltration of epoxy into the interface region was observed. The data presented in Table 5 regarding the mechanical properties of six composite sandwich types also supported digital imaging microscopic observations.

## 4. Conclusions

This study focused on investigating the mechanical behavior, including tensile, flexural, and interlaminar shear properties, of plasma-treated jute composite laminates. Additionally, the flexural behavior of bio-based jute fabric-reinforced sandwich composites was examined. Various composite laminates were fabricated, including non-woven mat fiber (MFC), jute fiber (JFC), dried jute fiber (DJFC), and plasma-treated jute fiber (TJFC). Furthermore, sandwich composites were constructed using jute fabric bio-based unsaturated polyester composite as the facing material and polyethylene terephthalate (PET70 and PET100), as well as polyvinyl chloride, as the core materials. The purpose was to compare their functional properties. The results revealed that plasma treatment of the jute composite laminate had a positive impact on several mechanical properties. This treatment resulted in a 14% improvement in Young’s modulus (reaching 7.17 GPa) and an 8.5% enhancement in tensile strength (reaching 53.61 MPa) compared to JFC. Similarly, the flexural strength (93.71 MPa) and flexural modulus (5.20 GPa) experienced a significant increase of 24% and 35%, respectively, compared to JFC. Furthermore, the findings indicated that incorporating PET100 foams as core materials in jute sandwich composites led to a substantial enhancement in their flexural properties. The microscopic images provided evidence of strong adhesion between the facings and PET100 as the core material in the jute fiber-reinforced sandwiches. This study demonstrated that these sandwiches have the potential to be utilized in a wide range of industrial applications as eco-friendly, cost-effective, and lightweight structures.

## Figures and Tables

**Table 1:** Properties of preparation of composite laminates.

Samples	Number of Layers	Thickness (mm)	Fiber Mass Fraction (%)
Mat fiber composite (MFC)	1 (2 *)	2.3 (4.9 *)	18
Jute fiber composite (JFC)	3 (7 *)	2.4 (4.9 *)	32
Dried jute fiber composite (DJFC)	3 (7 *)	2.4 (4.9 *)	32
Plasma-treated jute fiber composite (TJFC)	3 (7 *)	2.4 (4.9 *)	32

* In order to conduct the interlaminate shear test, it was necessary to have a specific thickness and number of layers in the laminates. To accommodate this requirement, a portion of the laminate was deliberately manufactured with a different thickness and number of layers (indicated by *) compared to the rest of the laminate.

**Table 2:** Tensile test result for the composite laminates.

Samples	Tensile Strength (MPa)	Tensile Strength SD (MPa)	Elongation to Break %	Elongation to Break SD %	Young’s Modulus (GPa)	Young’s Modulus SD (GPa)
Mat fiber composite (MFC)	35.66	1.92	4.43	0.46	3.63	0.19
Jute fiber composite (JFC)	49.07	2.80	4.70	0.55	6.12	0.61
Dried jute fiber composite (DJFC)	52.55	2.13	4.80	0.41	6.62	0.51
Plasma-treated jute fiber composite (TJFC)	53.61	2.38	4.70	0.32	7.17	0.88

**Table 3:** Flexural properties of the composite laminates.

Samples	Flexural Strength (MPa)	Flexural Strength SD (MPa)	Flexural Modulus (GPa)	Flexural Modulus SD (GPa)
Mat fiber composite (MFC)	71.26	7.21	4.12	0.71
Jute fiber composite (JFC)	70.78	3.98	3.38	0.32
Dried jute fiber composite (DJFC)	87.08	7.94	4.25	0.62
Plasma-treated jute fiber composite (TJFC)	93.71	7.46	5.20	0.73

**Table 4:** Interlaminar shear test properties of the jute fiber composites.

Samples	Short-Beam Strength (MPa)	Short-Beam Strength SD (MPa)	Max Force (N)	Max Force SD (N)
Mat fiber composite (MFC)	7.57	0.54	322.91	23.04
Jute fiber composite (JFC)	12.51	1.34	533.76	57.25
Dried jute fiber composite (DJFC)	14.11	0.51	601.88	21.66
Plasma-treated jute fiber composite (TJFC)	12.63	1.12	538.96	47.59

**Table 5:** Flexural properties of jute fiber sandwich panels.

Samples	Max Force (N)	Max Force SD (N)	Facing Ultimate Stress (FUS) (MPa)	FUS SD (MPa)	Core Shear Ultimate Stress (CSUS) (MPa)	CSUS SD (MPa)	Max Deflection (mm)	Max Deflection SD (mm)	Flexural Rigidity/Width (N.mm)
PET70	364.39	19.51	19.32	1.03	0.55	0.03	8.26	2.03	400,510
PET70-PLASMA	481.89	14.36	25.55	0.76	0.73	0.02	6.71	1.40	433,785
PET100-PLASMA	570.88	25.45	30.27	1.35	0.86	0.04	7.32	0.46	433,785
PVC	324.94	8.79	18.95	0.51	0.54	0.02	5.86	0.35	331,000
PVC-PLASMA	308.83	4.55	18.02	0.27	0.51	0.01	6.13	1.05	358,500
PET100	607.78	13.33	32.23	0.71	0.92	0.02	7.69	0.39	400,510

## Data Availability

The data presented in this study are available on request from the corresponding author.
